# Impact of Scaling and Periodontal Treatment during Pregnancy on the Risk of Adverse Birth Outcomes

**DOI:** 10.3390/jpm12020137

**Published:** 2022-01-20

**Authors:** Jhih-Jhen Chen, Dai-Rong Wu, Wei-Szu Lin, I-Chieh Chen, Jeng-Fen Liu, Hui-Ling Chen, Ching-Heng Lin

**Affiliations:** 1 Department of Dentistry, Taichung Veterans General Hospital, Taichung 40705, Taiwan; jen19901019@hotmail.com (J.-J.C.); dorina520201@gmail.com (D.-R.W.); 2 Department of Medical Research, Taichung Veterans General Hospital, Taichung 40705, Taiwan; weiszu@vghtc.gov.tw (W.-S.L.); icchen@vghtc.gov.tw (I.-C.C.); 3 Department of Pediatric Dentistry, Taichung Veterans General Hospital, Taichung 40705, Taiwan; jengliu@vghtc.gov.tw; 4 School of Dentistry, National Yang Ming Chiao Tung University, Taipei 11221, Taiwan; 5 Department of Health Care Management, National Taipei University of Nursing and Health Sciences, Taipei112303, Taiwan; 6 Department of Industrial Engineering and Enterprise Information, Tunghai University, Taichung 40705, Taiwan; 7 Department of Public Health, College of Medicine, Fu Jen Catholic University, New Taipei City 242062, Taiwan; 8 Institute of Public Health and Community Medicine Research Center, National Yang Ming Chiao Tung University, Taipei 11221, Taiwan; 9 Department of Medical Research, China Medical University Hospital, Taichung 40402, Taiwan

**Keywords:** adverse pregnancy outcomes, periodontal disease, low birth weight, scaling

## Abstract

Background: Adverse pregnancy outcomes (APOs) are associated with periodontal disease owing to the induction of a chronic systemic inflammatory response. Hence, knowledge of periodontal status during pregnancy is important in order to reduce the risk of APOs. The aim of this study was to compare the risk of APOs in women with and without periodontal disease to ascertain whether regular scaling performed prior to pregnancy improves the risk of APOs. Method: This case-control study enrolled1,386,887 pregnant women from the National Health Insurance Research Database who gave birth to their first child between 1 January 2004 and 31 December 2014. The study population included mothers who gave birth to low birth weight (LBW) and non-LBW newborns, totaling 86,958 and 1,299,929, respectively. Scaling and periodontal emergency treatment during and before pregnancy were assessed. Univariable and multivariable logistic regression analyses were performed to identify the associations between periodontal treatment and LBW risk. Results: Compared with the comparison cohort, the pregnant women who didnot have periodontal emergency treatment or scaling treatment during pregnancy exhibited a significantly increased risk of LBW than those who had treatment. Women who underwent scaling within the2 years before pregnancy or during pregnancy had a lower risk of delivering a LBW baby (odds ratio (OR), 0.93; 95% confidence interval (CI), 0.91–0.94). In the normal group, the mothers who had periodontal emergency treatment within the2 years before pregnancy or during pregnancy had a higher risk of delivering a LBW baby (OR, 1.05; 95% CI, 1.02–1.08). In those who had scaling treatment, a lower risk of delivering a LBW baby was noted (OR, 0.95; 95% CI, 0.93–0.97). Conclusion: The risk of LBW was significantly increased in women who underwent periodontal treatment, and our findings suggested that periodontal disease is an important risk factor for preterm LBW babies in an East Asian population.

## 1. Introduction

Periodontal disease is a serious infection of the gums that is responsible for a chronic inflammatory response in the body. It is mainly the result of infection and inflammation of the gums, which may lead to immunoinflammatory response activation [1]. According to the diagnostic criteria, the prevalence of periodontal disease in adults is about 10 to 60% [2]. Clinical manifestation of the disease is via the host immune response to periodontal pathogens, persistent inflammation, and the destruction of tooth-supporting connective tissue and bone [2]. This systemic inflammation has been found to be associated with adverse pregnancy outcomes (APOs), increasing the risk of APOs [3]. The pathophysiologic mechanism of a potential association between periodontal disease and APOs might be based on increased systemic inflammation caused by oral flora [4,5], which then influences the onset and course of APOs, including pre-eclampsia, low birth weight (LBW), and preterm delivery [2,4,6,7,8]. During pregnancy, approximately 40% pregnant women having periodontal disease [9]. Hence, confirmation of an independent association between periodontal disease and APOs would be of great public health importance, allowing prioritization of the development of preventive and therapeutic interventions to reduce the occurrence of APOs in pregnant women with periodontal disease. Several research studies have suggested that women with periodontal disease may be more likely to deliver premature or LBW babies than mothers with healthy gums [10,11,12]. LBW newborns may be at risk of long-term health problems, including intellectual and developmental disabilities, delayed motor skills, obesity, diabetes, high blood pressure and heart disease [13,14]. There are also similar complications for babies born before 37 weeks of pregnancy. In addition, other issues including respiratory problems, vision and hearing loss, or feeding and digestive problems are associated with preterm birth [15]. Recently, an association between periodontal disease and APOs has been inconsistently reported, with conflicting conclusions [3,16]. These results suggest that periodontal disease may have varying associations with the risk of APOs in different ethnicities and populations. Scaling and polishing removes deposits such as plaque and tartar from tooth surfaces. Over time, regular removal of these deposits may reduce the occurrence of gingivitis and prevent progression to periodontitis. According to the American Academy of Periodontology (AAP) and the European Federation of Periodontology (EFP), clinical recommendations indicate that non-surgical periodontal therapy could improve the periodontal condition and overall health of pregnant women [17]. Moreover, the American College of Obstetricians and Gynecologists also affirms the importance of oral health, having released a statement encouraging pregnant women to sustain their oral health and recommending regular dental cleanings during pregnancy [18].

Although some studies have demonstrated an association between periodontal disease and APOs, studies of Asian populations are rare. The purpose of this study was to determine the association between periodontal disease and APOs in a large Taiwanese national sample, obtained from the Taiwan National Health Insurance Research Database (NHIRD), to ascertain whether regular scaling before pregnancy or during pregnancy reduces the risk of APOs.

## 2. Materials and Methods

### 2.1. Ethical Statements

This retrospective case-control study was approved by the Institutional Review Board of Taichung Veterans General Hospital, Taiwan (IRB number CE19166A-2), and the study protocol was conducted in accordance with the Declaration of Helsinki. All available data were obtained from the Taiwan National Health Insurance Research Database (NHIRD), which collects information in a standardized format to fit researchers’ needs in different fields. Informed consent was not required, given that the claim database used in the present study contained only de-identified data. This study followed the Strengthening the Reporting of Observational Studies in Epidemiology (STROBE) reporting guideline.

### 2.2. Data Sources

This study was conducted using data from the NHIRD, which is maintained by the National Health Insurance program in Taiwan, which has operated since 1 March 1995, and enrolls 99.9% of Taiwan’s population. The program was established by Taiwan’s Ministry of Health and Welfare (MOHW) and collects information in a complete and standardized format to fit researchers’ needs in different fields.

### 2.3. Study Design

**Figure 1:**
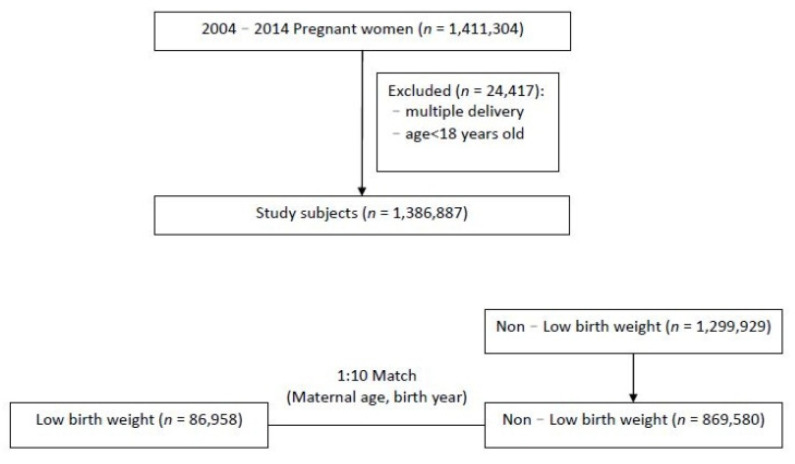
Flowchart illustrating the study design.

As shown in Figure 1, we selected women who gave birth to their first child between 1 January 2004 and 31 December 2014 as the study participants (*n* = 1,411,304); 24,417 women who had a maternal age of <18 years or a multiple pregnancy were excluded. The final study population included 1,386,887 women who gave birth to 86,958 and 1,299,929 LBW (birth weight < 2500 g) and non-LBW newborns, respectively. The non-LBW cohort was matched to the LBW cohort according to maternal age and year of birth at a ratio of 1 to 10. In total, 869,580 women who gave birth to non-LBW newborns comprised the comparison group. As periodontitis in the mother may affect birth weight, we attempted to confirm cases by identifying those who underwent scaling and periodontal emergency treatment during and before pregnancy.

### 2.4. Covariates

The covariates in our analysis included maternal age, income, urbanization of residence, model of delivery, maternal comorbidities and pregnancy-related complications. Diagnostic and procedural codes were classified according to the International Classification of Diseases, 9th revision, Clinical Modification (ICD-9-CM) coding system. Diabetes mellitus (DM, ICD-9-CM code 250), hypertension (ICD-9-CM code 401-405), hyperlipidemia (ICD-9-CM code 272), gestational DM (ICD-9-CM codes 648.0, 648.8 and 775.0), gestational hypertension (ICD-9-CM code 642.3), pre-eclampsia (ICD-9-CM codes 642.4, 642.5, 642.6 and 642.7), and placenta previa and abruptio placentae (ICD-9-CM codes 641, 762.0 and 762.1) were recorded. Hypertension, hyperlipidemia and DM were diagnosed before pregnancy.

### 2.5. Statistical Analysis

The demographic information is presented as mean ± standard deviation for continuous variables and number (percent) for categorical variables, and the Chi-squared test for categorical variables was used to compare data between the LBW group and the non-LBW control group. Univariable and multivariable logistic regression analyses were performed to estimate the associations between periodontal treatments and the risk of LBW. The odds ratios (ORs) and 95% confidence intervals (95% CIs) of variables in the two groups were calculated by logistic regression and adjusted according to the number of medical visits within two years prior to the index date. The association of periodontal treatment with the risk of LBW in all mothers was calculated and stratified analysis by maternal age was performed. All data were analyzed using SAS 9.4 software (SAS Institute Inc., Cary, NC, USA). Statistical significance was set at *p* = 0.05.

## 3. Results

A total of 956,538 pregnant women who were ≥18 years of age were identified in the present study; these women gave birth to 86,954 LBW and 869,540 normal weight babies (non-LBW, ≥2500 g—control group). The demographic characteristics are reported by categories of maternal age and maternal comorbidities in Table 1.

In contrast to the comparison cohort, the pregnant women who did not have periodontal emergency treatment or scaling treatment during pregnancy exhibited a significantly increased risk of LBW than those who had treatment. There were significant differences (*p* < 0.001) in income, urbanization of residence, model of delivery, maternal comorbidities, and pregnancy-related complications. Higher rates of cesarean section, diabetes mellitus, hypertension, and hyperlipidemia were observed in the LBW group.

Table 2 presents the results of univariable and multivariable logistic regression analyses of factors associated with LBW among the pregnant women who underwent periodontal treatment. Compared with the control group, the women who had scaling treatment within the 2 years before pregnancy or during pregnancy had a lower risk of delivering a LBW baby (OR,0.93; 95% CI, 0.91–0.94). Participants with a higher incomewere more likely to have a lower risk of delivering babies of LBW. In addition, the odds of LBW significantly increased with maternal comorbidities, especially hypertension (OR, 2.07; 95% CI, 1.95–2.20). The risk of LBW was significantly increased in participants with gestational hypertension (OR, 2.67; 95% CI, 2.51–2.84), pre-eclampsia or eclampsia (OR, 7.91; 95% CI, 7.65–8.18), and placenta previa and abruptio placentae (OR, 3.07; 95% CI, 2.98–3.15).

**Figure 2:**
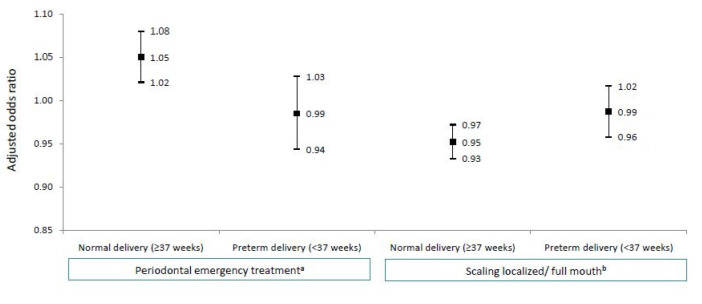
Multivariable logistic regression analysis of factors associated with low birth weight. The vertical lines represented the range of adjusted OR of each risk factors. ^a^ Mothers who have had periodontal emergency treatment within 2 years before pregnancy; ^b^ Mothers who have had localized scaling within 2 years before pregnancy.

As preterm birth is an important cause of LBW, we separated the women who had a normal delivery (≥37 weeks) from those who experienced a preterm birth (<37 weeks). As preterm birth and periodontal emergency treatment are important causes of LBW, we examined the risk of LBW in these groups. In the normal delivery group, the mothers who had periodontal emergency treatment within the2 years before pregnancy or during pregnancy had a higher risk of delivering a LBW baby (OR, 1.05; 95% CI, 1.02–1.08). For those who had scaling, a lower risk of delivering a LBW baby was noted (OR, 0.95; 95% CI, 0.93–0.97) (Figure 2).

## 4. Discussion

In our study, we observed an association between periodontal treatment and LBW. Pregnant women who did not have periodontal emergency treatment or scaling treatment during pregnancy exhibited a significantly increased risk of LBW than those who had treatment. These results indicated an association between the periodontal status of the mother and the risk of delivering a baby of LBW. Regular dental visits for scaling and oral hygiene education among pregnant woman were shown to be effective in reducing the rate of LBW, and consequently led to a better neonatal outcome [19]. The treatment prevalence in the non-LBW group was 21.2%, but was only 19.4% in the LBW group (OR, 0.94). Appropriate and timely scaling could lead to improved pregnancy outcomes and reduce the incidence of LBW. These results were consistent with those of a previous study that included 400 subjects, 200 of whom received periodontal therapy during pregnancy [20]. It was found in that study that the prevalence of preterm LBW (PLBW) was 1.84% in the treatment group and 10.11% in the control group. Moreover, Alves and Riberio reported an obvious association between periodontal disease and PLBW (OR, 8.9; 95% CI, 2.22–35.65) [21]. Jeffcot et al. reported similar results, and stated that the risk of having a LBW or preterm infant was increased by 4–7 times depending on the severity of periodontal disease [22]. Santos-Pereira et al. studied 124 Brazilian women, and identified a similar correlation between chronic periodontitis and LBW/preterm birth [23]. These studies produced consistent results, but had relatively small sample sizes, and the subjects did not include Asians. Our study showed periodontal disease to be a critical risk factor for LBW in an East Asian population using a large sample size. Recent studies have suggested that infection is a critical factor in APOs. Chronic periodontal infection could produce local/systemic host responses, leading to transient bacteremia. Up to 60% of cases of bacteremia are caused by Gram-negative species [24,25]. The cell wall components of gram-negative strains, such as the endotoxin lipopolysaccharide (LPS), are known to be potent inflammatory agents. LPS/endotoxin can gain access to gingival tissue, initiating and perpetuating local inflammatory reactions by inducing proinflammatory cytokine production; they also play a central role in particular cell death processes through translocation between the gut, blood and other tissues. The activation of inflammation responses and cytokine cascades could play an important role in the pathogenic process of preterm labor, LBW, and pre-eclampsia in pregnant women [8,26]. These subgingival bacteria and proinflammatory cytokines from inflamed periodontal tissue could enter the bloodstream, reach the maternal–fetal interface, trigger or worsen maternal inflammation, and increase the levels of prostaglandin and cytokines, such as interleukin-1β or tumor necrosis factor-α (TNF-α) [27,28,29]. Thus, it appears that periodontal disease may play a nonspecific role in various APOs [2]. The potential biological mechanisms behind the association between maternal periodontal health and APOs has been proposed to trigger inflammation and the suppression of insulin-like growth factor 2 (IGF-2) in the fetal–placental unit [30]. IGF-2 is a peptide hormone that plays a key role in regulating cell proliferation, growth, migration, differentiation and apoptosis [31], showing that IGF-2 is a critical regulating factor in fetal growth and embryo development. Two major pathways (direct and indirect) have been reported [30]. In the direct pathway, oral microorganisms and their components reach the fetal–placental unit and induce an immune response via hematogenous dissemination, or dental bacteria reach the placenta through an ascending route via the genitourinary tract. In the indirect pathway, the inflammatory mediators locally produced in periodontal tissues, such as prostaglandin E2 and TNF-α, circulate and impact the fetal–placental unit. Alternatively, during periodontitis, inflammatory mediators and/or microbial components diffuse systemically from the oral cavity to the liver, enhancing cytokine production (e.g., IL-6) and acute phase protein responses (e.g., CRP), which then impact the fetal–placental unit [30]. Premature birth and LBW have great impacts in terms of newborn health, the economy, society and family. Treating LBW infants requires high levels of healthcare resources and may pose a heavy burden on parents. Previous studies have shown that LBW newborns are more prone to illness such as neuromotor abnormalities, lung diseases or cerebral palsy, and LBW is frequently related to child morbidity and mortality [32]. Furthermore, it is also a significant factor associated with long-term physical and mental deficiencies, and problems related to behavior, learning, and psychosocial improvements during childhood [32]. In this study, we determined that periodontal disease is an important risk factor for preterm LBW babies, and simple scaling treatment was shown to reduce the risk of LBW in an East Asian population. This therapy involves no risk, can be carried out easily, and is relatively inexpensive. These results suggested that scaling treatment could be advantageous if introduced systematically in prenatal care. As a result of our findings, a special dentistry unit should be opened at Obstetrics and Gynecology Departments to treat pregnant women who attend for check-ups. Where a special unit cannot be created, a good communication platform between obstetrician, gynecologist and dentist should be established. Obstetricians and gynecologists should suggest that pregnant women go to the dentist for a dental examination and scaling treatment every 3 months during pregnancy, and that those preparing for pregnancy undergo regular 6-month scaling [19].

There are several strengths of our study that are worthy of note. First, up to 99.99% of Taiwan’s population is enrolled under the National Health Insurance program [33]. Thus, the NHIRD, derived from data from the NHI, exemplifies a population-level data source for the generation of real-world evidence to support clinical decisions. We used this database to conduct this nationwide, population-based cohort study. Selection bias was avoided in our investigation of the risk of LBW in mothers with periodontitis of different ages who underwent specific treatments, with the outcome being defined according to physician diagnoses. Second, complete information on outpatient appointments, inpatient stays, and prescriptions was included in the NHIRD, which is maintained by the National Health Insurance program in Taiwan, has operated since 1 March 1995, and enrolls 99.9% of Taiwan’s population of 23.69 million, meaning that medical visits were not under-reported. Finally, at the population level, more than 98% of the population in Taiwan are of Chinese Han ethnicity; thus, this ensured that race was not a confounder.

However, there were some limitations of our study. First of all, we may have underestimated the prevalence of periodontitis in the pregnant women as we only collected data on those who attended for therapy; therefore, the data we gathered only represented patients who underwent treatment during or before their pregnancy. There may have been patients with periodontitis who did not attend for dental care. Second, the information was obtained from the Taiwan NHIRD; the results therefore may not apply to the entire Asian population, or to the whole world, as Taiwan is a developed country with a sound health insurance system. Third, subjects with current use or a history of tobacco and alcohol use were not excluded from this study. Smoking and alcohol intake are known risk factors for PLBW; however, the prevalence of smoking and alcohol-drinking is relatively low among Taiwanese pregnant woman.

## 5. Conclusions

In conclusion, we conducted a retrospective case-control study using a population-based database of Taiwanese subjects, and determined that periodontal disease is an additional important risk factor for preterm LBW babies our findings suggested that regular dental visits for scaling treatment and oral hygiene education in pregnant woman reduced the rate of LBW, and consequently resulted in a better neonatal outcome.

## Figures and Tables

**Table 1:** Basic characteristics of the study subjects.

Characteristic	Non-LBW	LBW	*p* Value
	(*n* = 869,580) (%) ^a^	(*n* = 86,958) (%) ^a^	
Maternal age (years) (%)			
<25	160,880 (18.5)	16,088 (18.5)	
25–34	564,270 (64.9)	56,427 (64.9)	
≥35	144,430 (16.6)	14,443 (16.6)	
Income (TWD) (%)			<0.001
≤15,840	148,445 (17.1)	17,616 (20.3)	
15,841–28,800	435,297 (50.1)	42,982 (49.4)	
28,801–45,800	201,303 (23.1)	18,986 (21.8)	
>45,800	84,535 (9.7)	7374 (8.5)	
Urbanization of residence (%)			<0.001
Urban	529,899 (61)	51,571 (59.3)	
Suburban	111,103 (12.8)	11,537 (13.3)	
Rural	228,062 (26.2)	23,805 (27.4)	
Mode of delivery (%)			<0.001
Vaginal delivery	574,754 (66.1)	51,866 (59.6)	
Cesarean section	294,826 (33.9)	35,092 (40.4)	
Maternal comorbidity (%)			<0.001
Diabetes mellitus	5741 (0.7)	895 (1)	
Hypertension	5595 (0.6)	1972 (2.3)	
Hyperlipidemia	10,698 (1.2)	1577 (1.8)	
Pregnancy-related complication (%)			
Gestational diabetes mellitus	17,366 (2)	2049 (2.4)	<0.001
Gestational hypertension	4557 (0.5)	1532 (1.8)	<0.001
Pre-eclampsia or eclampsia	8881 (1)	7315 (8.4)	<0.001
Placenta previa and abruptio placentae	27,030 (3.1)	7867 (9)	<0.001

^a^ Comparisons of categorical variables were analyzed using the Chi-square test.

**Table 2:** Multivariate analysis of factors associated with low birth weight.

Variables	OR	95% CI		*p* Value ^a^	OR	95% CI		*p* Value ^a^
Procedures (during–within 2 years before pregnancy)								
Periodontal emergency treatment	-	-	-	-	1.00	0.98	1.03	0.673
Scaling localized/full mouth	0.93	0.91	0.94	<0.001	0.93	0.91	0.94	<0.001
Maternal age								
<25	1.00 (reference)				1.00 (reference)			
25–34	1.00	0.98	1.02	0.977	1.00	0.98	1.02	0.980
≥35	0.89	0.87	0.91	<0.001	0.89	0.87	0.91	<0.001
Income								
≤15,840	1.00 (reference)				1.00 (reference)			
15,841–28,800	0.84	0.82	0.85	<0.001	0.84	0.82	0.85	<0.001
28,801–45,800	0.81	0.79	0.82	<0.001	0.81	0.79	0.82	<0.001
>45,800	0.75	0.73	0.77	<0.001	0.75	0.73	0.77	<0.001
Urbanization of residence								
Urban	1.00 (reference)				1.00 (reference)			
Suburban	1.04	1.02	1.07	<0.001	1.04	1.02	1.07	<0.001
Rural	1.04	1.03	1.06	<0.001	1.04	1.03	1.06	<0.001
Mode of delivery								
Vaginal delivery	1.00 (reference)				1.00 (reference)			
Cesarean section	1.06	1.04	1.07	<0.001	1.06	1.04	1.07	<0.001
Maternal comorbidity								
Diabetes mellitus	0.94	0.86	1.01	0.106	0.94	0.86	1.01	0.105
Hypertension	2.07	1.95	2.20	<0.001	2.07	1.95	2.20	<0.001
Hyperlipidemia	1.09	1.02	1.15	0.008	1.09	1.02	1.15	0.008
Pregnancy-related complication								
Gestational diabetes mellitus	0.99	0.95	1.04	0.757	0.99	0.95	1.04	0.756
Gestational hypertension	2.67	2.51	2.84	<0.001	2.67	2.51	2.84	<0.001
Pre-eclampsia or eclampsia	7.91	7.65	8.18	<0.001	7.91	7.65	8.18	<0.001
Placenta previa and abruptio placentae	3.07	2.98	3.15	<0.001	3.07	2.98	3.15	<0.001

^a^ The models were adjusted for individuals’ age, income, and maternal comorbidity.

## Data Availability

Raw data for this work were obtained by application from the National Health Insurance Research Database, Taiwan (http://nhird.nhri.org.tw/en/index.html, accessed on 19 January 2022), and may not be shared according to the database’s rules governing use. Access to the data used in this study may be obtained by citizens of Taiwan who fulfill the requirements of conducting research projects. The data used in this study cannot be made available in the manuscript, the supplemental files, or in a public repository due to the Personal Information Protection Act executed by Taiwan’s government, starting in 2012. Requests for data can be sent as a formal proposal to obtain approval from the ethics review committee of the appropriate governmental department in Taiwan.
